# Comparative Analysis of Septin Modifiers, Forchlorfenuron and UR214‐9, on Mitochondrial Fragmentation and Lytic Cell Death

**DOI:** 10.1002/cm.70013

**Published:** 2025-07-30

**Authors:** Dominik Brokatzky, Rajdeep Das, Hannah Painter, Rakesh K. Singh, Serge Mostowy

**Affiliations:** ^1^ Department of Infection Biology London School of Hygiene & Tropical Medicine London UK; ^2^ Institut Pasteur, Université Paris Cité, CNRS UMR3691 Dynamics of Host‐Pathogen Interactions Unit Paris France; ^3^ Department of Obstetrics and Gynecology University of Rochester Medical Center Rochester New York USA

**Keywords:** cell death, macrophages, septin modifiers, septins, zebrafish

## Abstract

Septins are conserved GTP‐binding proteins that play key roles in cell division, mitochondrial dynamics and immune responses. Despite their importance to human health, pharmacological compounds to modify septins remain limited. Forchlorfenuron (FCF) was the first small molecule identified to modify septins, disrupting their organisation and promoting mitochondrial fragmentation. A more potent FCF analog (UR214‐9) has recently been developed, but its effects on mitochondria were unknown. Here, we compare FCF and UR214‐9 in vitro using macrophages and in vivo using zebrafish larvae. We demonstrate that both modifiers induce mitochondrial fragmentation in macrophages without altering mitochondrial mass or SEPT7 expression. Consistent with mitochondrial fragmentation, both modifiers trigger lytic cell death in a dose‐dependent manner following lipopolysaccharide (LPS) priming. In vivo, both modifiers exhibit dose‐dependent effects on the survival of zebrafish larvae, although UR214‐9 was significantly more toxic. In agreement with in vitro results, we observed that FCF induces macrophage cell death and caspase‐1 activity in zebrafish larvae. Together, our findings show that both septin modifiers impact mitochondrial integrity and macrophage survival. Understanding how septin modifiers regulate immune responses may have important implications for inflammatory disease research and could lead to the development of septin‐based medicines for conditions characterised by dysregulated inflammation.

## 1 Introduction

Septins are a conserved family of GTP‐binding proteins (including SEPT2, SEPT6, SEPT7 and SEPT9) that assemble into hetero‐oligomeric complexes and play fundamental roles in cell division and immune responses (Mostowy and Cossart 2012). Septins have recently been implicated in the regulation of inflammatory pathways and cell death (Brokatzky et al. 2024; Mazon‐Moya et al. 2017; Van Ngo et al. 2022). Despite these functions critical for human health, there is a significant lack of pharmacological compounds available to specifically manipulate septin biology.

One of the first small molecules discovered to interact with septins was forchlorfenuron (FCF), a synthetic plant cytokinin used in agriculture to promote fruit enlargement (Reynolds et al. 1992). FCF functions by binding to and competitively inhibiting a cytokinin dehydrogenase, increasing intracellular cytokinin levels (Kopečný et al. 2010). The role of FCF in plant biology is well characterised, but its effects on septin biology and mammalian cells are poorly understood.

Work in yeast ( *Saccharomyces cerevisiae* ) has shown that FCF treatment led to growth inhibition and formation of abnormal septin structures (Iwase et al. 2004), as well as mitochondrial fragmentation (Heasley et al. 2014). Notably, these effects were rapidly reversible, and no alterations in other cytoskeletal elements were observed, suggesting that FCF is specific for septins. Work using human epithelial cells has shown that FCF alters SEPT2/6/7 assembly, inducing the formation of thicker parallel filaments and promoting cytokinesis defects (Hu et al. 2008). More recently, FCF has been found to activate the NLRP3 inflammasome (a key component of pyroptosis, a pro‐inflammatory form of lytic cell death) in macrophages through a mitochondrial signalling axis (Holley et al. 2024). Lytic cell death (including pyroptosis) is characterised by the release of inflammatory cytokines and cell rupture, and plays a crucial role in the immune response to infections and tissue damage (Brokatzky and Mostowy 2022; Place and Kanneganti 2019). In the case of FCF, treatment results in loss of mitochondrial membrane potential (MMP) and ablates mitochondrial respiration, suggesting broader implications for septin modifiers in cellular metabolism and immune responses. The discovery of FCF as a septin‐modifying compound has elevated septin biology research, for example, recent work showing roles for septins in cell blebbing and cancer metastasis (Weems et al. 2023), but its use is viewed as limited because of off‐target effects (Heasley and McMurray 2016; Heasley et al. 2014).

Efforts have been made to develop compounds alternative to FCF, with improved efficacy and specificity, to modify septin biology. One such molecule is UR214‐9 (an analog of FCF) that also inhibits septin GTPase activity but presents increased efficiency of GTPase inhibition as compared to FCF (Kim et al. 2020; Zhovmer et al. 2024). Thus, compared to FCF, UR214‐9 can be viewed as a more potent inhibitor of septin dynamics (Kim et al. 2020).

**FIGURE 1:**
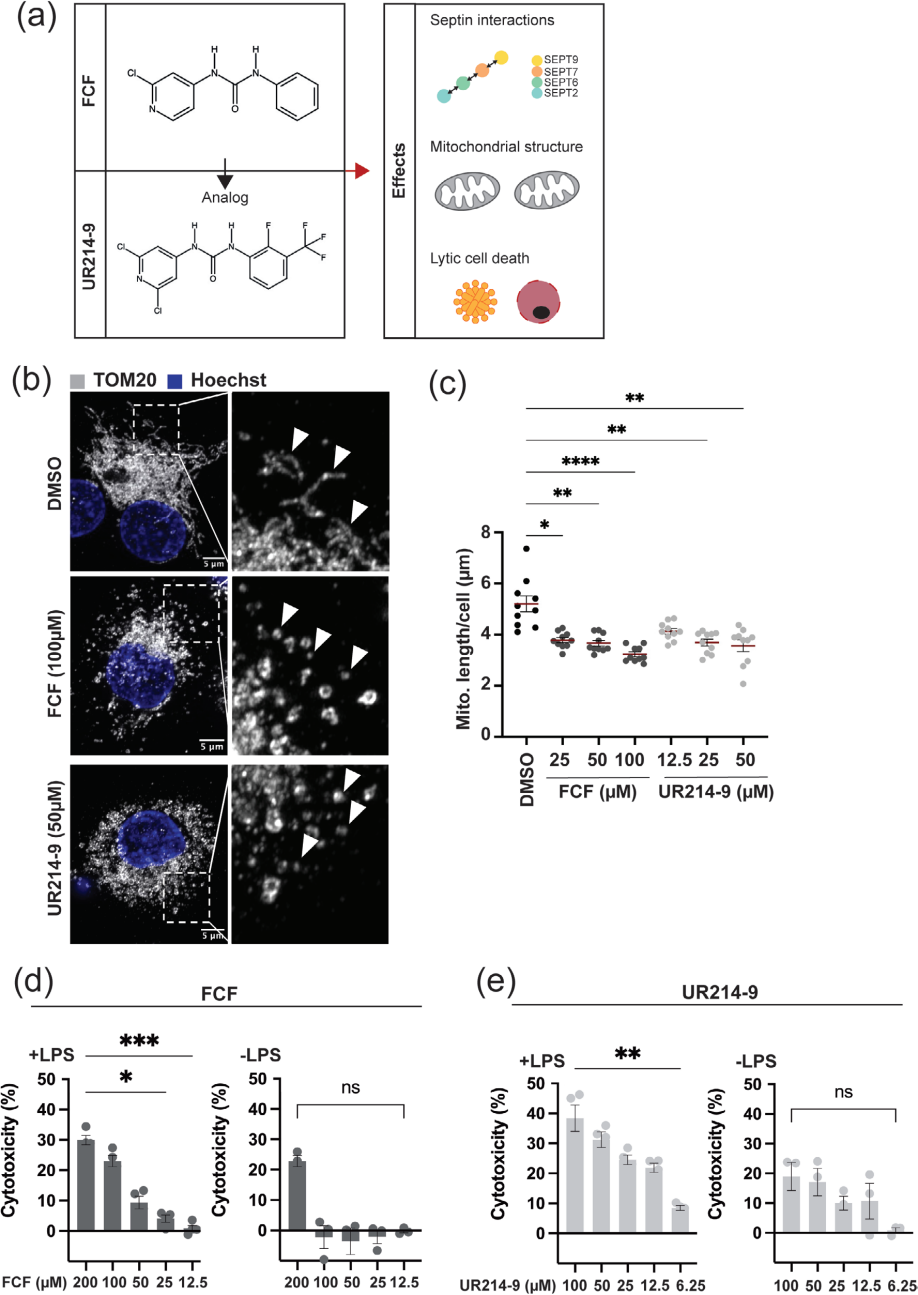
FCF and UR214‐9 induce mitochondrial fragmentation and lytic cell death in THP‐1 cells. Graphical representation of FCF and UR214‐9 and its known effects on cells. Representative images of mitochondria (TOM20, Alexa488) showing fragmentation post 2 h treatment with FCF or UR214‐9. Analysis of mitochondrial fragmentation post FCF (25, 50,100 μM) and UR214‐9 (12.5, 25, 50 μM) treatment for 2 h at the indicated concentration. Data were tested for statistical significance using the non‐parametric Mann–Whitney test. ns = *p* > 0.05, * = *p* < 0.05, ** = *p* < 0.01, *** = *p* < 0.001, **** = *p* < 0.0001. LDH release assay demonstrating dose‐dependent cytotoxicity post 2 h treatment with FCF with indicated concentrations and with or without LPS (100 nM, 2 h) treatment. Data (*N* > 3) tested for statistical significance using the non‐parametric Mann–Whitney test. ns = *p* > 0.05, * = *p* < 0.05, ** = *p* < 0.01, *** = *p* < 0.001. LDH release assay demonstrating dose‐dependent cytotoxicity post 2‐h treatment with UR214‐9 at indicated concentrations and with or without LPS (100 nM, 2 h) treatment. Data (*N* > 3) tested for statistical significance using the non‐parametric Mann–Whitney test. ns = *p* > 0.05, * = *p* < 0.05, ** = *p* < 0.01, *** = *p* < 0.001.

In this study, we compared FCF and UR214‐9 in vitro using macrophages and in vivo using zebrafish ( *Danio rerio* ) larvae, testing for mitochondrial fragmentation and macrophage cell death (Figure 1a). In the case of macrophages, we showed that both compounds induce mitochondrial fragmentation and trigger lytic cell death in a dose‐dependent manner. In the case of zebrafish larvae, we showed that FCF induces macrophage cell death and caspase‐1 activity. We conclude that both FCF and UR214‐9 promote mitochondrial fragmentation and macrophage survival, but UR214‐9 is more cytotoxic.

## 2 Results

### 2.1 Septin Modifiers Induce Mitochondrial Fragmentation

Previous work using human and mouse macrophages has shown that FCF activates the NLRP3 inflammasome via mitochondrial fragmentation, and that FCF leads to loss of MMP resulting in lytic cell death (Holley et al. 2024). We hypothesised that UR214‐9 also induces mitochondrial fragmentation and lytic cell death. To test this, we treated THP‐1‐derived macrophages with FCF and UR214‐9 and measured structural differences in the mitochondrial network. Mitochondria were visualised by high‐resolution confocal microscopy using immunostaining for TOM20, a marker of the outer mitochondrial membrane (OMM). We observed that both compounds induced mitochondrial fragmentation, as evidenced by a shift from long, interconnected networks to smaller, punctate structures (Figure 1b). In line with this, quantitative microscopy showed a significant decrease in mitochondrial length for both FCF‐ and UR214‐9‐treated macrophages using three different concentrations (25, 50, 100 μM for FCF and 12.5, 25, 50 μM for UR214‐9), as compared to DMSO‐treated macrophages (Figure 1c).

To test if fragmented mitochondria are targeted to degradation, we measured mitochondrial mass via flow cytometry analysis using TOM20 immunostaining. Previous reports (using microscopy) demonstrated that loss of MMP in FCF‐treated cells did not affect TOM20 intensity (Holley et al. 2024). In agreement, we did not detect a change in TOM20 intensity in FCF‐treated cells, as compared to DMSO‐treated cells, indicating that mitochondrial mass is unaffected (Figure S1a, Gating strategy Figure S1d). We observed similar results for UR214‐9‐treated cells (Figure S1a). These findings suggest that mitochondrial fragmentation is not resulting in mitochondrial degradation, but rather in reorganisation of mitochondrial networks.

To test septin levels in macrophages treated with septin inhibitors, we next analysed expression of SEPT7 in macrophages by flow cytometry. Under these experimental conditions, our analysis showed that neither FCF nor UR214‐9 affected SEPT7 expression (Figure S1b, Gating strategy Figure S1d). Previous work showed that FCF treatment promoted the formation of long, stable septin fibers and stabilized septin structures in *Ashbya gossypii* (DeMay et al. 2010; Hu et al. 2008). Therefore, we treated macrophages with FCF and UR214‐9 and visualized SEPT7 via immunostaining (Figure S1e). We did not observe significant changes in septin assemblies (e.g., filaments associated to the plasma membrane) under experimental conditions used here.

Together, these results show that both FCF and UR214‐9 induce mitochondrial fragmentation in macrophages, without affecting mitochondrial mass or SEPT7 protein levels.

### 2.2 Septin Modifiers Induce Lytic Cell Death

To assess whether UR214‐9 triggers lytic cell death in a manner comparable to FCF (Holley et al. 2024), we performed a lactate dehydrogenase (LDH) release assay, a commonly used assay to quantify cell membrane rupture and cytotoxicity. Both FCF and UR214‐9 caused significant LDH release in LPS‐stimulated macrophages (Figure 1d,e). Results were dose‐dependent, with higher concentrations of compounds leading to greater cytotoxicity (Figure 1d,e). Notably, FCF demonstrates lower toxicity than UR214‐9, as indicated by its dose‐dependent killing (cytotoxicity of 10%: 25 μM for FCF compared to 6.25 μM for UR214‐9).

Lytic cell death is often associated with inflammasome activation (Brokatzky and Mostowy 2022; Cookson and Brennan 2001; Galluzzi et al. 2018). We showed that UR214‐9 induces caspase‐1 activity, indicating inflammasome activation (Figure S1c), consistent with previous work reporting that FCF induces inflammasome activation (Holley et al. 2024). Next, we tested whether lipopolysaccharide (LPS) priming is essential for the induction of cell death. Our results show that LPS priming is essential for the induction of cell death in both FCF and UR214‐9‐treated cells (Figure 1d,e), indicating that both septin‐modifying compounds induce lytic cell death.

### 2.3 Toxicity In Vivo Differs Between FCF and UR214‐9

Zebrafish serve as an excellent model for studying the effects of chemical compounds in vivo (Cassar et al. 2020). Zebrafish are also well established for research on innate immunity and lymphocyte behaviour during inflammatory processes and cell death (Brokatzky et al. 2024; Cassar et al. 2020; Gomes and Mostowy 2020; Gomes et al. 2023; Torraca et al. 2023). Using a zebrafish infection model, we recently found that inhibiting cell death during *Shigella* infection (known to trigger pyroptosis in macrophages) reduces survival (Brokatzky et al. 2024). In this case, pyroptosis induction and zebrafish survival were septin dependent (Brokatzky et al. 2024).

**FIGURE 2:**
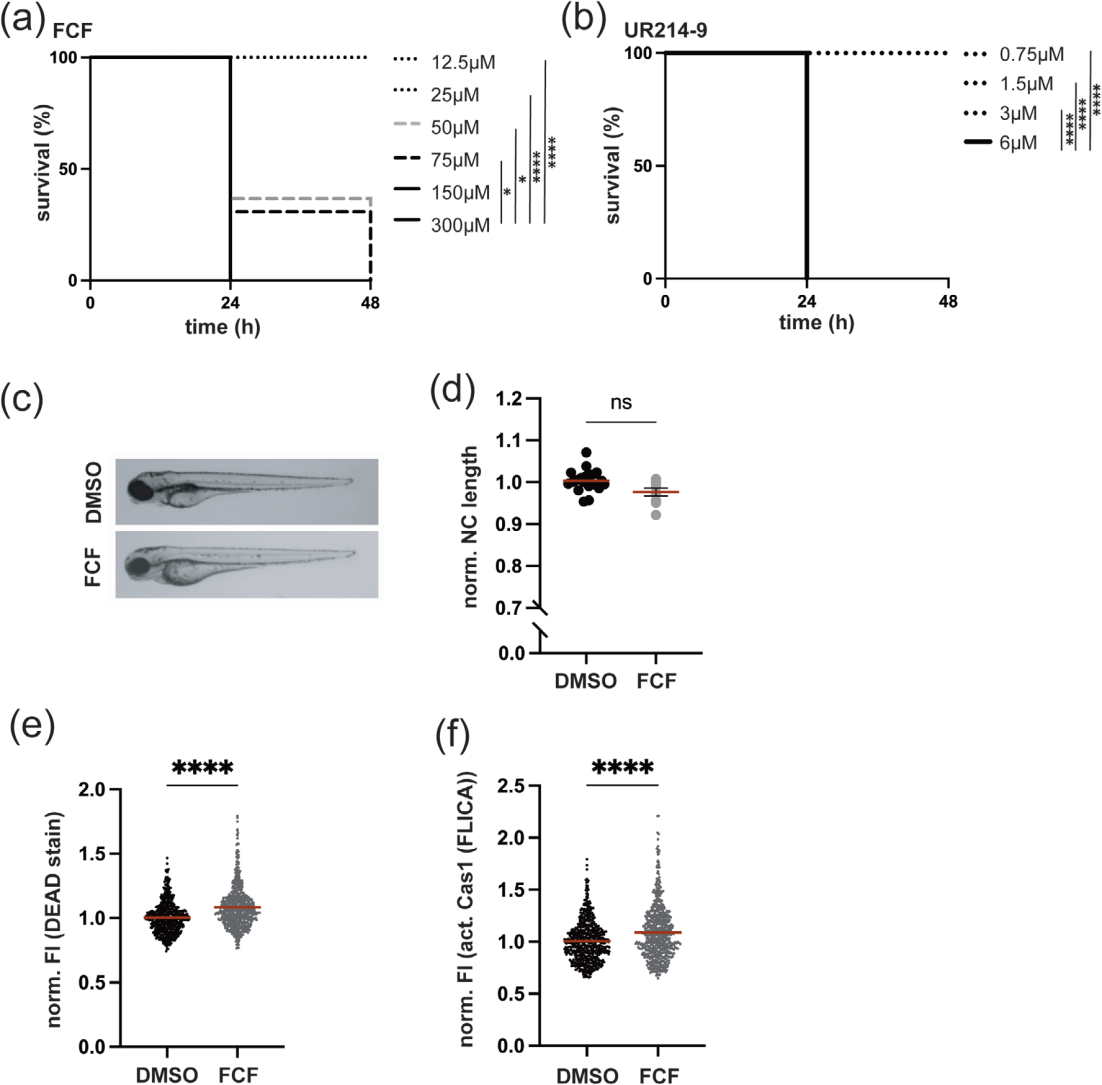
Toxicity of FCF or UR214‐9 on macrophages in vivo. Survival curves of zebrafish larvae exposed to different concentrations of FCF and monitored for 48 h. *N* = 3 with > 10 larvae per experiment. *Indicated significance compared to 300 μM FCF treatment. Survival curves of zebrafish larvae exposed to different concentrations of UR214‐9 and monitored for 48 h. *N* = 3 with > 10 larvae per experiment. *Indicated significance compared to 6 μM UR214‐9 treatment. Representative image of DMSO or FCF (25 μM) treated larvae 24 h post treatment to monitor larvae development. DMSO and FCF (25 μM) treated larvae 24 h post treatment were analysed by measuring the length of the notochord (NC) of individual larvae. Data (*N* = 3) are shown as normalised to the length of NC to DMSO treated larvae. Data were tested for statistical significance using the non‐parametric Mann–Whitney test. ns = *p* > 0.05. Flow cytometry analysis of macrophages of FCF (25 μM, 24 h) treated cells to detect dead macrophages. Data tested for statistical significance using the non‐parametric Mann–Whitney test. ns = *p* > 0.05, * = *p* < 0.05, ** = *p* < 0.01, *** = *p* < 0.001, **** = *p* < 0.0001. Flow cytometry analysis of macrophages of FCF (25 μM, 24 h) treated cells to detect caspase‐1 activity in macrophages. Data were tested for statistical significance using the non‐parametric Mann–Whitney test. ns = *p* > 0.05, * = *p* < 0.05, ** = *p* < 0.01, *** = *p* < 0.001, **** = *p* < 0.0001.

Septin modifiers FCF and UR214‐9 triggered lytic cell death in THP‐1 macrophages (Figure 1). To investigate if FCF and UR214‐9 triggered lytic cell death in vivo, we performed experiments in zebrafish larvae. To first evaluate the in vivo toxicity of FCF and UR214‐9, zebrafish larvae at 2 days post‐fertilisation (dpf) were exposed to increasing concentrations of either compound, where development and survival were monitored over time. Both FCF and UR214‐9 exhibited toxicity in a concentration‐dependent manner, with higher doses leading to significant mortality (Figure 2a,b), while DMSO‐treated larvae did not show reduced survival (Figure S2a). Notably, FCF demonstrates lower toxicity than UR214‐9 (as indicated by its non‐lethal dose of 25 μM compared to 3 μM for UR214‐9). This difference suggests that UR214‐9 has a much stronger effect on cell survival, consistent with results obtained in vitro using macrophages (Figure 1d,e). Based on these findings, we selected a concentration of FCF and UR214‐9 that did not impair zebrafish development or survival for subsequent experiments (Figure 2c,d and Figure S2b).

To investigate the effects of FCF and UR214‐9 on macrophages in vivo, we used flow cytometry. Cell death was analysed using Live/Dead and caspase‐1 activity staining, alongside transgenic zebrafish *Tg(mpeg1::Gal4‐FF)*gl25/*Tg(UAS: LIFEACT‐GFP)*mu271 or *Tg(mpeg1::Gal4‐FF)*gl25/*Tg(UAS‐E1b::nfsB.mCherry)*c264 larvae expressing macrophage‐specific fluorescent markers (Gating strategy Figure S2e–g). FCF‐treated larvae exhibited significantly increased macrophage cell death and caspase‐1 activity, as compared to DMSO‐treated larvae (Figure 2e,f), in agreement with results from THP‐1 survival experiments (Figure 1d). Under these experimental conditions, UR214‐9 treatment had no significant impact on macrophage survival in vivo (Figure S2c,d).

## 3 Concluding Remarks

Our study provides new insights into the impact of FCF and UR214‐9 on mitochondrial integrity and macrophage survival. Both septin modifiers induced mitochondrial fragmentation in THP‐1 macrophages without affecting mitochondrial mass. These results are consistent with studies showing that septins interact with mitochondrial fission machinery and are located at the mitochondrial constriction site (Mageswaran et al. 2023; Pagliuso et al. 2016; Sirianni et al. 2016). Although we assume that compounds act through septins, the precise mechanisms by which FCF and UR214‐9 induce mitochondrial fragmentation are not yet clear.

A key finding of our study is the induction of macrophage lytic cell death by septin modifiers. Both FCF and UR214‐9 triggered dose‐dependent cytotoxicity, which was enhanced by LPS priming (suggesting activation of pyroptosis). Inflammasome activation has previously been shown for FCF treatment (Holley et al. 2024); here we show caspase‐1 activity alongside FCF‐mediated macrophage cell death in vivo. We also reveal in vivo limitations of using UR214‐9, consistent with previous reports of UR214‐9 being more cytotoxic in epithelial cells (Kim et al. 2020).

We provide evidence that UR214‐9 is more efficient than FCF, as highlighted by the lower concentration of UR214‐9 necessary to induce mitochondrial fragmentation and lytic cell death. Using zebrafish larvae for toxicity screening, we show that both compounds caused dose‐dependent killing. Although FCF is less toxic than UR214‐9, the reasons for this are not yet known, and intermediate concentrations of UR214‐9 could potentially impact macrophages in vivo (though it remains below detection under experimental conditions used here).

In summary, we investigated the role of septin modifiers in regulating mitochondrial integrity and macrophage survival in vitro using THP‐1 cells and in vivo using zebrafish. Future work is required to explore underlying mechanisms and treatment impact on mitochondrial functions (including Seahorse assays), and to more fully enable the potential of septin‐targeting compounds as therapeutic agents for diseases characterised by dysregulated inflammation (such as autoimmune diseases and chronic infections). It will also be exciting to test future septin‐based medicines, including ReS19‐T (Princen et al. 2024), using experimental pipelines we describe here.

## 4 Materials and Methods

### 4.1 THP‐1 Cell Culture and Differentiation

THP‐1 monocyte cells (ATCC, CAT#TIB‐202) were cultured in RPMI‐1640 medium (ThermoFisher Scientific, CAT#11875093), supplemented with 10% (v/v) heat‐inactivated fetal bovine serum (hi‐FBS) (Gibco, CAT#10500‐064), under standard incubation conditions (37°C, 5% CO_2_). For macrophage differentiation, cells were exposed to 100 nM phorbol 12‐myristate 13‐acetate (PMA) (Sigma‐Aldrich, CAT#P8139) in RPMI‐1640 with 10% (v/v) hi‐FBS for 48 h, followed by a recovery period of 24 h in fresh RPMI containing 10% (v/v) hi‐FBS.

### 4.2 Confocal Microscopy

THP‐1 cells (1 × 10^5^) were seeded in 8‐well μ‐slides (IBIDI) and differentiated prior to fixation with 4% (v/v) paraformaldehyde (PFA) (ThermoFisher Scientific, CAT#28908) in PBS for 15 min. Cells were then washed three times with PBS and permeabilized using 0.3% (v/v) Triton X‐100 in PBS for 5 min. After another triple PBS wash, cells were blocked in 1% (w/v) BSA (Sigma‐Aldrich, CAT#A9647) with 0.1% (v/v) Triton X‐100 for 30 min. Primary antibodies (Septin7 Anti‐human Rabbit IgG (Tecan (IBL), CAT#18991); Tom20 antibody (F10) (Santa Cruz, CAT#sc‐17 764)) were applied in blocking solution and incubated overnight at 4°C. After washing, secondary antibodies conjugated to fluorophores (Goat anti‐Mouse IgG (H + L) Highly Cross‐Adsorbed Secondary Antibody, Alexa Fluor 647 (ThermoFisher Scientific, CAT#A‐21236); Goat anti‐Rabbit IgG (H + L) Highly Cross‐Adsorbed Secondary Antibody, Alexa Fluor 488 (ThermoFisher Scientific, CAT#A‐11034)) were added for 1 h at room temperature (RT). Hoechst (ThermoFisher Scientific, CAT#H3570) was used for nuclear staining for 15 min at RT, followed by ethanol washes. Slides were mounted with IBIDI mounting medium and stored at 4°C until imaging. Images were captured on a Zeiss LSM 880 confocal microscope using a 63×/1.4 NA C‐Plan Apo oil objective and processed with ZEN Black (v2.3), applying Airyscan features.

### 4.3 Flow Cytometry of THP‐1 Cells

Differentiated macrophages were treated with specific septin‐targeting compounds. Following treatment, cells were detached using trypsin, washed with RPMI + 10% hi‐FBS, and then rinsed three times in PBS. After centrifugation (800 × g, 5 min), cells were fixed in 4% PFA for 15 min at RT. Permeabilization was achieved using 100% methanol stored overnight at −20°C. Cells were stained for 2 h at RT with primary antibodies (Septin7 Anti‐human Rabbit IgG (Tecan (IBL), CAT#18991); Tom20 antibody (F10) (Santa Cruz, CAT#sc‐17 764)) diluted in PBS containing 1% BSA and 0.3% Triton X‐100. After two PBS washes, secondary AlexaFluor‐conjugated antibodies (Alexa Fluor 488 (ThermoFisher Scientific, CAT#A‐11034)) were applied for 1 h at RT. Single‐cell data acquisition was performed on a Cytek Aurora cytometer, and results were analyzed using FlowJo software v10.8.1.

### 4.4 LDH Cytotoxicity Assay

Cell death was assessed using the CyQUANT LDH Cytotoxicity Assay Kit (ThermoFisher Scientific, CAT#C20301), following the manufacturer's protocol. A total of 2.5 × 10^4^ cells/well were seeded in 96‐well plates and differentiated accordingly. Treatment with LPS (Merck, CAT#L4391‐1MG) and septin inhibitors or modulators was done as specified in figure legends. Readouts were obtained using a SpectraMax iD5 plate reader.

### 4.5 Detection of Caspase‐1 Activation

Caspase‐Glo 1 Inflammasome Assay (Promega, CAT#G995) was used to detect caspase‐1 activity in the supernatant of UR214‐9 treated differentiated THP‐1 macrophages, following the manufacturer's protocol.

### 4.6 Mitochondrial Morphology Analysis

Mitochondrial lengths were quantified using the Mitochondria Analyzer plugin in Fiji (ImageJ). *Z*‐stacked confocal images (0.2 μm intervals) were processed to generate maximum intensity projections and binarized using thresholding. The plugin was then employed to measure mitochondrial length per cell.

### 4.7 Zebrafish Husbandry

Embryos were derived from naturally mating adult zebrafish and maintained at 28.5°C in 0.5% E2 or E3 embryo media supplemented with 0.3 mg/mL methylene blue. Transgenic lines utilized included Tg(*mpeg1::Gal4‐FF*)gl25/Tg(*UAS: LIFEACT‐GFP*)mu271 and Tg(*mpeg1::Gal4‐FF*)gl25/Tg(*UAS‐E1b::nfsB.mCherry*)c264. For experimental treatments, larvae were incubated in E2 or E3 media at 28.5°C for 48–72 h.

### 4.8 Chemical Treatment in Zebrafish

Larvae were immediately exposed to septin‐targeting drugs in E3 medium upon initiation of treatment. Control groups were kept in E3 with an equivalent concentration of DMSO. All treatments were carried out in the dark at 28.5°C for 24 or 48 h, depending on the experiment.

### 4.9 Flow Cytometry and Caspase‐1 Detection in Zebrafish

For flow cytometry analysis of zebrafish macrophages, we modified a previously described method (Brokatzky et al. 2024). Briefly, 20–30 larvae at 2 days post‐fertilization (dpf) from either Tg(*mpeg1::Gal4‐FF*)gl25/Tg(*UAS: LIFEACT‐GFP*)mu271 or Tg(*mpeg1::Gal4‐FF*)gl25/Tg(*UAS‐E1b::nfsB.mCherry*)c264 lines were processed following treatment. Larvae were dissociated using 4% trypsin for 15 min at 28.5°C, centrifuged (800 × g, 5 min), washed in PBS, and filtered through 4‐mm strainers. Cells were then stained in 300 μL FLICA 660 Caspase‐1 (Immunochemistry, CAT#9122) detection reagent and LIVE/DEAD fixable violet stain (1:1000) for 1 h at RT in darkness. After PBS washes and fixation in 4% PFA at 4°C overnight, samples were analyzed the following day using Cytek Aurora, and data were processed with FlowJo v10.8.1.

### 4.10 Statistics and Data Analysis

GraphPad Prism 10.0 was used for all statistical evaluations. Specific details are provided in the figure legends. Data are expressed as mean ± SEM from at least three independent replicates per condition. Non‐parametric tests (e.g., Kruskal–Wallis or Mann–Whitney U) were used as appropriate, with *p*‐values < 0.05 considered statistically significant. Survival analysis of zebrafish larvae was performed using the Log‐rank (Mantel‐Cox) test.

## Author Contributions

S.M. conceived and supervised this study. D.B., R.D., H.P., R.K.S., and S.M. designed the experiments. D.B. and R.D. performed experiments using tissue culture cells. D.B. and H.P. performed experiments using zebrafish. All authors performed data analysis and took part in the interpretation of results and preparation of materials for the manuscript. D.B. and S.M. wrote the manuscript with comments from all authors.

## Ethics Statement

All animal procedures adhered to the Animals (Scientific Procedures) Act 1986 and were authorized by the UK Home Office under project licenses PPL P4E664E3C and PP5900632.

## Conflicts of Interest

The authors declare no conflicts of interest.

## Supporting information


**FIGURE S1.** Related to.
(a) Flow cytometry analysis of differentiated THP‐1 macrophages for FCF or UR214‐9 treated cells (2 h) to detect mitochondrial mass (TOM20 staining). Data (*N = 3*) tested for statistical significance using non‐parametric Mann–Whitney test. ns = *p >* 0.05.
(b) Flow cytometry analysis of differentiated THP‐1 macrophages for FCF or UR214‐9 treated cells (2 h) to detect SEPT7 expression levels. Data (*N = 3*) tested for statistical significance using non‐parametric Mann–Whitney test. ns = *p* > 0.05.
(c) Analysis of LPS primed (2 h) differentiated THP‐1 macrophages for UR214‐9 treated cells (2 h) to detect caspase‐1 activity, using Caspase‐Glo 1 Inflammasome Assay. Data (*N = 3*) tested for statistical significance using non‐parametric Mann–Whitney test. * = *p* < 0.05.
(d) Gating strategy for TOM20 and SEPT7 staining.
(e) Immunostaining of SEPT7 (grey) and Hoechst (blue) in DMSO, FCF or UR214‐9 treated differentiated THP‐1 macrophages, 2 h post treatment. Scale bar 10 μm.

**FIGURE S2.** Related to.
(a) Survival curves of zebrafish larvae exposed to DMSO and monitored for 48 h. *N* = 3 with > 10 larvae per experiment.
(b) Representative image of DMSO or UR215‐9 (3 μM) treated larvae 24 h post treatment, to monitor larvae development. DMSO or UR215‐9 (3 μM) treated larvae 24 h post treatment was analysed by measuring the length of the notochord (NC) of individual larvae. Data (*N* = 3) are shown as normalised to the length of NC to DMSO treated larvae. Data tested for statistical significance using non‐parametric Mann–Whitney test. ns = *p* > 0.05.
(c) Flow cytometry analysis of macrophages of UR215‐9 (3 μM, 24 h) treated cells to detect dead macrophages. Data tested for statistical significance using non‐parametric Mann–Whitney test. ns = *p* > 0.05.
(d) Flow cytometry analysis of macrophages of UR215‐9 (3 μM, 24 h) treated cells to detect caspase‐1 activity in macrophages. Data tested for statistical significance using non‐parametric Mann–Whitney test. ns = *p* > 0.05.
(e) Gating strategy for macrophage detection in dissociated *Tg*(*mpeg1::Gal4‐FF*)gl25/*Tg*(*UAS‐E1b::nfsB.mCherry*)c264 larvae.
(f) Representative histogram of Live/Dead stain and caspase‐1 activity staining (FLICA), of zebrafish macrophages.

## Data Availability

The data that support the findings of this study are available from the corresponding author upon reasonable request.
