# Supplementary figures and images for: Comparative Analysis of Septin Modifiers, Forchlorfenuron and UR214‐9, on Mitochondrial Fragmentation and Lytic Cell Death
Source: Cytoskeleton (Hoboken). 2025 Jul 30;83(4):179–86. doi: 10.1002/cm.70013 (PMC13080067; doi:10.1002/cm.70013)

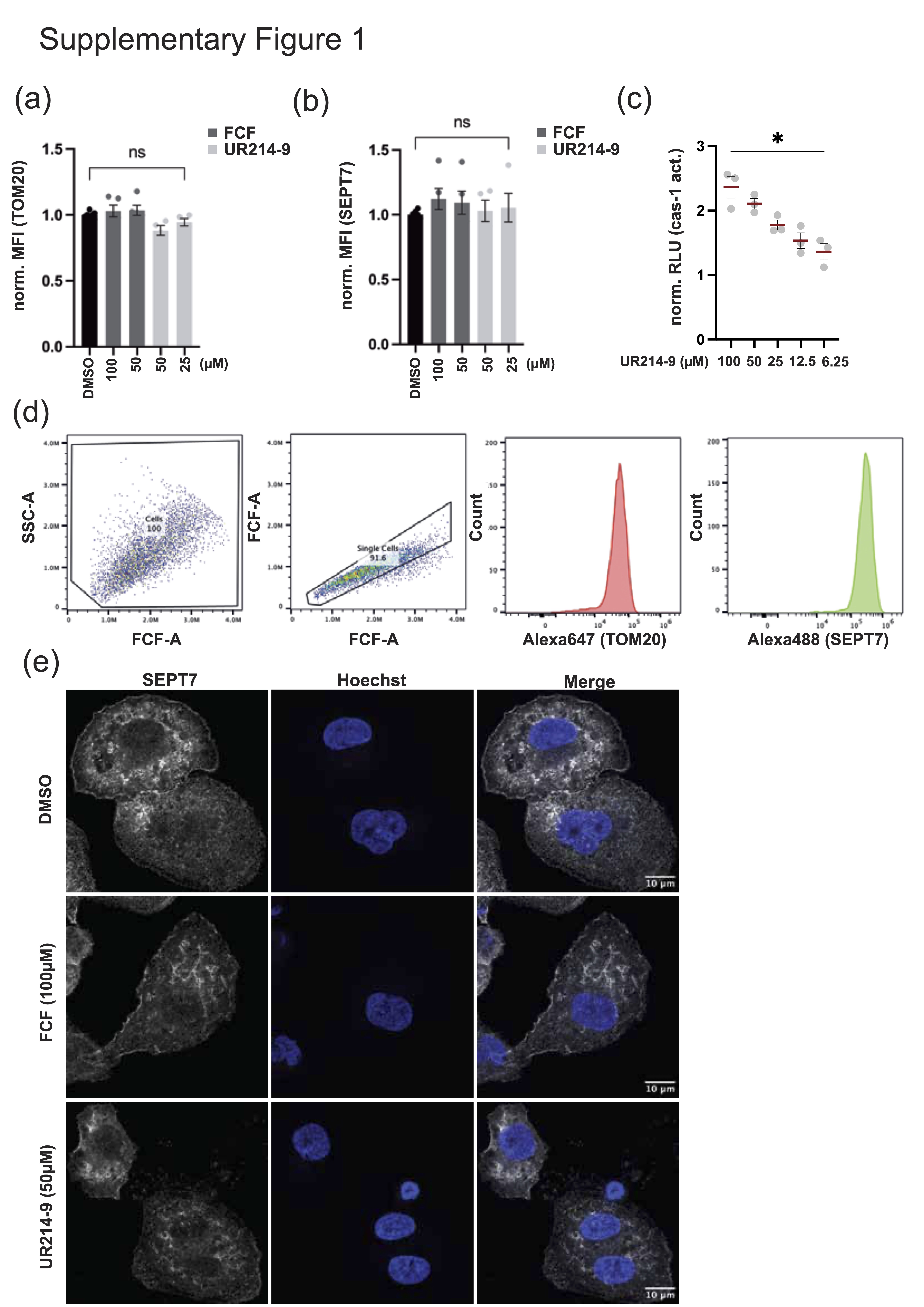

Supplement: Supplementary file 1 — FIGURE S1. Related to. (a) Flow cytometry analysis of differentiated THP‐1 macrophages for FCF or UR214‐9 treated cells (2 h) to detect mitochondrial mass (TOM20 staining). Data (N = 3) tested for statistical significance using non‐parametric Mann–Whitney test. ns = p > 0.05. (b) Flow cytometry analysis of differentiated THP‐1 macrophages for FCF or UR214‐9 treated cells (2 h) to detect SEPT7 expression levels. Data (N = 3) tested for statistical significance using non‐parametric Mann–Whitney test. ns = p > 0.05. (c) Analysis of LPS primed (2 h) differentiated THP‐1 macrophages for UR214‐9 treated cells (2 h) to detect caspase‐1 activity, using Caspase‐Glo 1 Inflammasome Assay. Data (N = 3) tested for statistical significance using non‐parametric Mann–Whitney test. * = p < 0.05. (d) Gating strategy for TOM20 and SEPT7 staining. (e) Immunostaining of SEPT7 (grey) and Hoechst (blue) in DMSO, FCF or UR214‐9 treated differentiated THP‐1 macrophages, 2 h post treatment. Scale bar 10 μm. [file CM-83-179-s002.jpg]

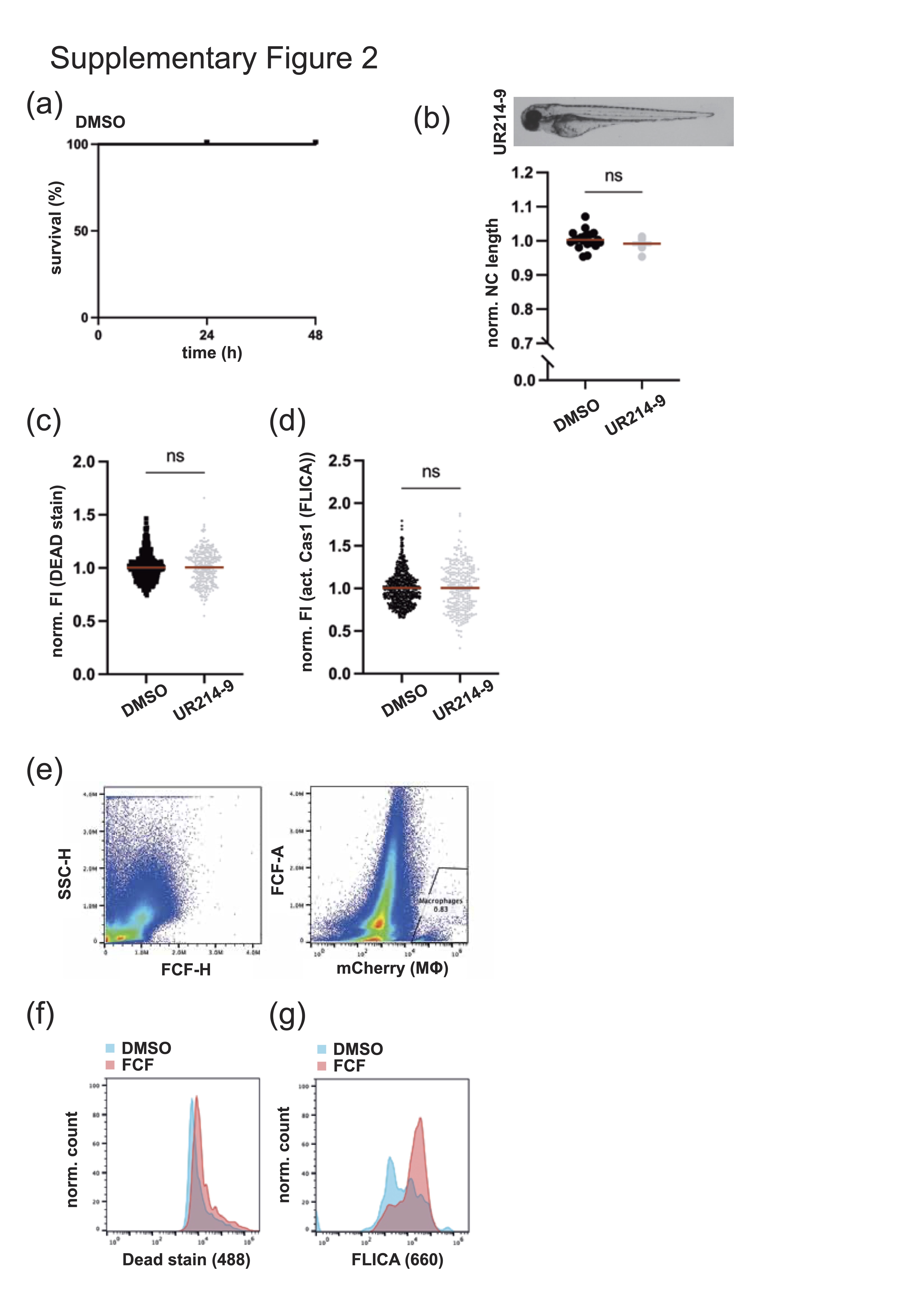

Supplement: Supplementary file 2 — FIGURE S2. Related to. (a) Survival curves of zebrafish larvae exposed to DMSO and monitored for 48 h. N = 3 with > 10 larvae per experiment. (b) Representative image of DMSO or UR215‐9 (3 μM) treated larvae 24 h post treatment, to monitor larvae development. DMSO or UR215‐9 (3 μM) treated larvae 24 h post treatment was analysed by measuring the length of the notochord (NC) of individual larvae. Data (N = 3) are shown as normalised to the length of NC to DMSO treated larvae. Data tested for statistical significance using non‐parametric Mann–Whitney test. ns = p > 0.05. (c) Flow cytometry analysis of macrophages of UR215‐9 (3 μM, 24 h) treated cells to detect dead macrophages. Data tested for statistical significance using non‐parametric Mann–Whitney test. ns = p > 0.05. (d) Flow cytometry analysis of macrophages of UR215‐9 (3 μM, 24 h) treated cells to detect caspase‐1 activity in macrophages. Data tested for statistical significance using non‐parametric Mann–Whitney test. ns = p > 0.05. (e) Gating strategy for macrophage detection in dissociated Tg(mpeg1::Gal4‐FF)gl25/Tg(UAS‐E1b::nfsB.mCherry)c264 larvae. (f) Representative histogram of Live/Dead stain and caspase‐1 activity staining (FLICA), of zebrafish macrophages. [file CM-83-179-s001.jpg]
